# Vertebral artery mobilization for C1–2 reduction and fixation

**DOI:** 10.3171/2020.1.FocusVid.19555

**Published:** 2020-01-01

**Authors:** Michael M. McDowell, Andrew Venteicher, Ezequiel Goldschmidt, Maximiliano Nuñez, David O. Okonkwo, Paul A. Gardner

**Affiliations:** Department of Neurological Surgery, University of Pittsburgh Medical Center, Pittsburgh, Pennsylvania

**Keywords:** vertebral artery mobilization, Goel’s technique, atlantoaxial dissociation, video

## Abstract

Craniocervical instability due to chronic atlantoaxial dissociation presents the challenge of providing adequate decompression, reduction, and fixation to promote long-term stability while avoiding iatrogenic vertebral artery dissection or entrapment. The authors present one patient with chronic atlantoaxial dissociation and basilar invagination treated via Goel’s technique and with bilateral vertebral artery mobilization. There was substantial decompression and reduction postoperatively and the patient was discharged with a stable examination. Vertebral artery mobilization at the C1–2 junction can be safely performed via a standard midline suboccipital incision and dissection without vertebral artery injury.

The video can be found here: https://youtu.be/VS1Mt1dBLO4.

## Transcript

**0:20** This is a case in which bilateral vertebral artery mobilization during operative reduction of basilar invagination was performed using Goel’s technique.1

The patient is a 56-year-old woman with rheumatoid arthritis who presented to clinic with upper extremity numbness, shocking neck pain with movement, and progressive upper extremity weakness.

**FIG. 1.**
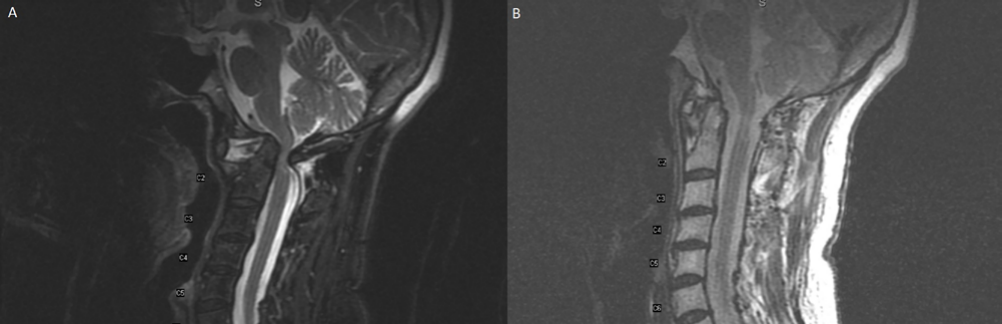
**A:** Preoperative T2-weighted sagittal MR image demonstrating severe craniocervical junction canal stenosis secondary to basilar invagination from C1–2 joint subluxation. **B:** Postoperative T2-weighted sagittal MR image demonstrating dramatic decompression of craniocervical junction and C1–2 reduction supported by C1–3 instrumented fixation.

**0:42** Her cervical MRI demonstrated severe cervicomedullary junction compression and basilar invagination, as seen here on axial and sagittal T2 sequences (Fig. 1). A subsequent CTA of the neck demonstrated severe chronic atlantoaxial subluxation with worrisome displacement of the vertebral arteries bilaterally.

On examination the patient was pathologically hyperreflexic and had profound myelopathy. A C1–2 decompression with reduction and instrumentation using the Goel technique was offered. Bilateral vertebral artery mobilization was planned prior to cervical manipulation.

**1:24** The patient was pinned at the superior temporal line above the pinna bilaterally. She was placed in prone position and her head was secured in a slight military flex position in order to facilitate exposure and screw placement. A standard midline incision was made from just below the inion to below the C2 spinous process and the craniocervical junction was exposed via dissection through the median raphe.

**FIG. 2.**
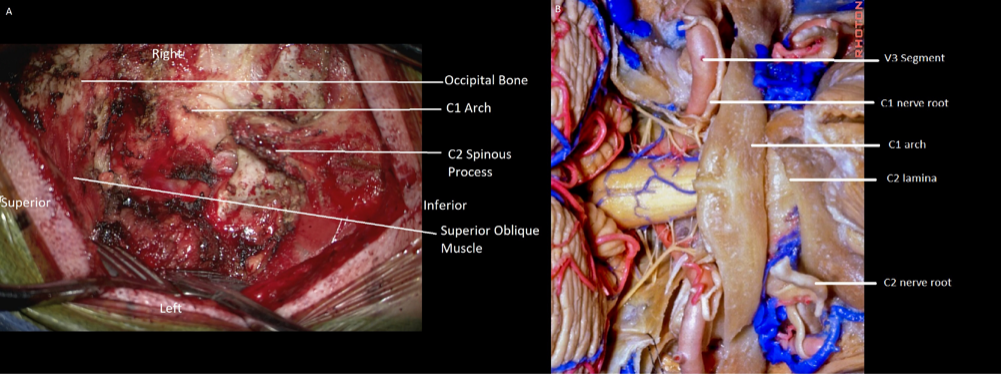
Intraoperative **(A)** and cadaveric **(B)** dissection depicting the anatomy of the posterior craniocervical junction. Panel B © 2019, Rhoton Collection.

**1:49** Here is an anatomic depiction of the V3 segment in relation to C1 and C2 from a midline perspective. From a more lateral perspective the relationship of the ascending V3 segment between C1 and C2 is appreciated, as is its close relationship to the C1 and C2 nerve roots (Fig. 2).

**2:10** After the initial midline exposure the C1 arch is dissected laterally via a combination of blunt dissection and electrocautery. Medial aspects of the suboccipital musculature such as the superior oblique muscle may be dissected in order to provide a wider exposure. The superior margin of the C1 arch is identified and the transverse process of C1 is then defined. Next exposure of the C2 lateral mass is performed in a similar manner to the C1 exposure, with frequent use of Doppler localization of the vertebral artery to avoid iatrogenic injury.

**2:52** The C1 nerve root is identified and sharply transected to allow for greater access to and mobilization of the looped V3 segment entering the transverse foramen, which is visualized during gentle blunt dissection. The C2 nerve root is similarly cauterized and transected in order to give access to the vertical segment of V3. This is a key step anytime the ascending segment of the vertebral artery must be localized, as well as being an important landmark for identification of the C1–2 joint.

**3:25** The superior aspect of the lateral mass of C2 is dissected and the foramen transversarium is identified. A periosteal dissection of the bone adjacent to the vertebra artery is performed using a Penfield one. Further dissection between the vertebral artery and the PLL is performed to identify the eroded joint space. The procedure is performed again on the right side with exposure of C1 and C2, and dissection of the ascending vertebral artery and associated nerve roots.

**4:00** The anatomic relationship of the vertebral artery to C1 and C2 is demonstrated in this figure.

**4:05** Here the hypermobility of the unstable C1–2 joint is demonstrated bilaterally and the vertebral arteries are seen nicely isolated from the joint complex.

Decompression via a C1 laminectomy and instrumentation was then performed. C1 lateral mass screws were placed with 5° of medial trajectory and bicortical purchase. At C2 bilateral pedicle screws were placed. At C3 lateral mass screws were placed due to poor bone quality.

**4:36** The C1–C2 joints were bilaterally decorticated. Titanium interfacet spacers with bone allograft were inserted at C1–C2, inducing distraction of the joints and some joint reduction. An assistant proceeded to untether the Mayfield clamp from the bed and neutral alignment of the head over the cervical spine was achieved via head extension. This configuration was locked into place with rods, which further contributed to joint reduction. Fluoroscopic imaging confirmed reduction as defined by the relationship of the C1 anterior tubercle with respect to the C2 vertebral body.

**5:16** A multilayer fascial closure with dissolvable sutures followed by skin staples was performed. The patient remained at her neurological baseline and was ultimately discharged home. While not always necessary, vertebral artery mobilization is helpful in situations where manipulation of vertebral alignment may stress the vertebral arteries, or where dissection or drilling of deeper structures is impaired by their positioning. This may be magnified in situations where aberrant vessel location inhibits safe spinal instrumentation.
